# Neuroimaging of Adult Gliomas: An Update

**DOI:** 10.5334/jbr-btr.1415

**Published:** 2017-11-18

**Authors:** Niloufar Sadeghi

**Affiliations:** 1 Hôpital Erasme, Brussels, BE

**Keywords:** Glioma, MRI, Brain, fMRI, diffusion, perfusion, Imaging

Brain gliomas are the most common primary malignant brain tumors in adults and are graded according to the World Health Organization (WHO), from grade I to grade IV, with increasing malignancy. Recent studies in the field of molecular markers and genomic alterations conducted to a new molecular-based classification of gliomas [1]. First it is important for a radiologist to be aware of the latest modifications of the pathological classification for brain gliomas according to their genomic characterization. In fact, gliomas with the same morphological histology show different responses to treatment, depending on the presence or absence of a specific gene mutation and molecular marker. Gliomas have been classified based on their cellular morphology to astrocytomas, oligodendrogliomas, and mixed oligoastrocytomas. According to the new classification, diffuse gliomas are characterized depending on the presence of molecular markers, such as 1p19q codeletion, mutational status of isocitrat dehydrogenase (IDH), telomerase reverse transcriptase (TERT), and tumor protein p53 (TP53) genes. TERT mutations are diagnostic of oligodendrogliomas (with 1p19q codeletion) or glioblastoma (without 1p19q codeletion); TP53 mutations in the presence of IDH mutation are diagnostic of astrocytomas. Therefore, according to the new classification, at the molecular level, mixed oligoastrocytomas do not exist.

Molecular markers also provide prognostic information. A 1p19q codeletion is predictive of favorable prognostic and sensitivity to chemotherapy, and methylation of the methylguanine methytransferase (MGMT) promoter gene in glioblastomas is predictive of a better outcome after adjuvant chemotherapy. Also, IDH-mutant grades II and III diffuse gliomas have a more favorable prognosis than those with IDH gene wild type (IDHwt). In parallel, the advances in functional and physiologic magnetic resonance (MR) imaging techniques along with anatomic MR imaging allows us to not only improve diagnostic specificity but also to precisely localize the lesion, define the best surgical approach, and finally perform a longitudinal follow-up after surgery to assess the response to a specific treatment [2].

**Figure 1:**
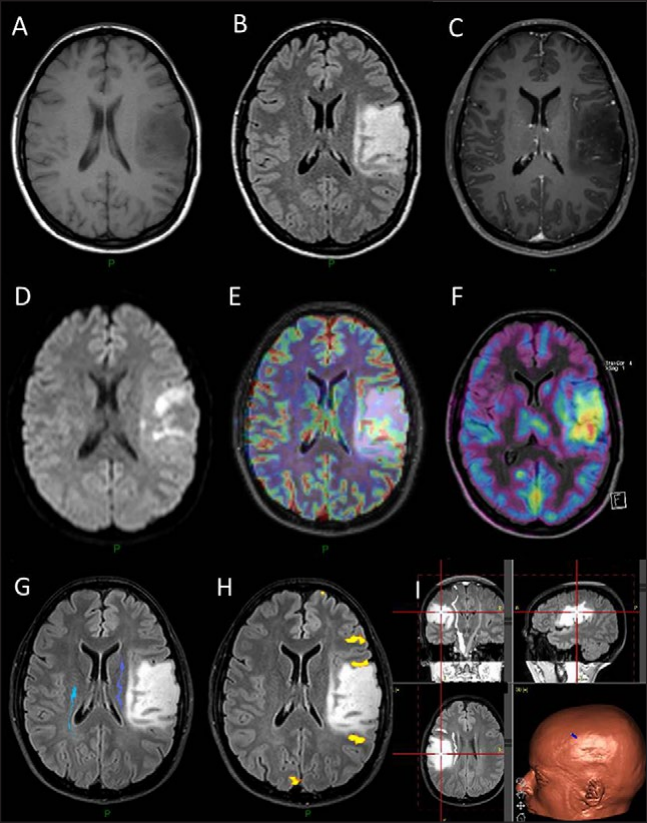
27-year-old woman presenting with headache. **A–C**: Anatomic MR imaging, including T1 (A), FLAIR (B), and T1 with contrast (C), show an intra-axial tumor in the left fronto-temporal region which do not show any enhancement. **D–F**: Physiologic and metabolic imaging, including diffusion (D), perfusion (E), and PET-methionine (F), respectively, show diffusion-restricted areas in the tumor with slight hypervascularisation and areas of methionine uptake. **G–I**: Tractography-based on DTI (G) as well as language fMRI (H) have been used to surgically remove the tumor by means of the neuronavigation system (I).

In this presentation, we will review the role of different anatomic and advanced nonanatomic imaging techniques in the management of brain gliomas. In patients with a suspected brain tumor, anatomic imaging should include T1, T2, and fluid attenuation inversion recovery (FLAIR) MR sequences and T1 with contrast. Computed tomography (CT) may also be helpful by easily detecting calcifications and hemorrhage. Diffusion- and perfusion-weighted images are currently widely used and improve the diagnostic specificity by differentiation between different tumor subtypes and identifying signs of higher malignancy Figure 1. Furthermore, perfusion imaging may also provide prognostic information independently of histological tumor grade. There is a hypothesis that the biological behavior of tumors with different molecular markers may show different imaging features, and, therefore, some studies are currently focused on finding correlations between imaging features and glioma molecular markers to obtain a noninvasive test and a better selection of patients who should undergo surgery [3]. Functional MRI (fMRI) and diffusion tensor imaging (DTI) are used in the preoperative planning of tumors located close to a functional cortex Figure 1. The use of metabolic imaging modalities such as MR spectroscopy (MRS) and positron emission tomography (PET) is less defined and depends on the local clinical experience and availability.
